# Mapping bacterial diversity and metabolic functionality of the human respiratory tract microbiome

**DOI:** 10.1080/20002297.2022.2051336

**Published:** 2022-03-16

**Authors:** Leonardo Mancabelli, Christian Milani, Federico Fontana, Gabriele Andrea Lugli, Chiara Tarracchini, Francesca Turroni, Douwe van Sinderen, Marco Ventura

**Affiliations:** a Laboratory of Probiogenomics, Department of Chemistry, Life Sciences and Environmental Sustainability, University of Parma, Parma, Italy; b Interdepartmental Research Centre “Microbiome Research Hub”, University of Parma, Parma, Italy; c APC Microbiome Institute and School of Microbiology, Bioscience Institute, National University of Ireland, Cork, Ireland

**Keywords:** Human respiratory tract, oral microbiota, pulmonary, microbiome, shotgun metagenomics, sputum

## Abstract

**Background:**

The Human Respiratory Tract (HRT) is colonized by various microbial taxa, known as HRT microbiota, in a manner that is indicative of mutualistic interaction between such microorganisms and their host.

**Aim:**

To investigate the microbial composition of the HRT and its possible correlation with the different compartments of the respiratory tract.

**Methods:**

In the current study, we performed an in-depth meta‐analysis of 849 HRT samples from public shotgun metagenomic datasets obtained through several distinct collection methods.

**Results:**

The statistical robustness provided by this meta-analysis allowed the identification of 13 possible HRT-specific Community State Types (CSTs), which appear to be specific to each anatomical region of the respiratory tract. Furthermore, functional characterization of the metagenomic datasets revealed specific microbial metabolic features correlating with the different compartments of the respiratory tract.

**Conclusion:**

The meta-analysis here performed suggested that the variable presence of certain bacterial species seems to be linked to a location-related abundance gradient in the HRT and seems to be characterized by a specific microbial metabolic capability.

## Introduction

The human body harbors a large number of microorganisms that live in a close symbiotic relationship with their host, constituting a complex ecological community known as the microbiota [1]. An increasing number of studies have reported on the roles exerted by the human microbiota in maintaining physiological homeostasis of the host [2,3], modulating immunological [4,5], metabolic [6,7], and nutritional [8–10] functionalities. Moreover, specific microbiota compositions have been identified for particular anatomical body sites, including the gastrointestinal tract [11–13], the skin [14,15], vagina [16,17], and the respiratory tract [18,19]. Interestingly, for a long time, the Human Respiratory Tract (HRT) had erroneously been considered microbiologically sterile; it was modern metagenomic approaches through which in depth investigations of HRT’s microbial communities have been made possible [18,20].

The HRT can be divided into the Upper Respiratory Tract (URT), which includes the nasal cavity, pharynx, and larynx, and the Lower Respiratory Tract (LRT), which comprises the trachea, the primary bronchi, and lungs [18]. Many studies assessing the HRT microbiota have focused on specific regions, i.e. URT or LRT, with their associated microbiota composition being delineated using distinct sampling approaches, such as nasal [21–23] or lung lavage fluid [24,25], local swabs [26,27], biopsies [26,28] or sputum [29–31].

Microbiota-based studies targeting healthy individuals based on culture-independent methods has revealed that the LRT microbiota shares microbial colonizers with the URT microbiota [32,33] and that the oropharynx appeared to be the major bacterial source of the whole lung microbiota in adults [32,34]. Furthermore, culture-independent approaches, e.g. 16S rRNA gene sequencing, facilitated disentanglement of the complexity of microbial communities at genus-level resolution, revealing that the HRT microbiota composition is dominated by members of the *Prevotella, Streptococcus, Veillonella, Pseudomonas, Fusobacterium, Haemophilus*, and *Neisseria* genera [20,35]. Besides, relationships between microbiota composition and respiratory diseases were investigated, disclosing a low load and higher biodiversity of bacterial communities in healthy subjects when compared to those associated with certain diseases, some of which not being directly associated with bacterial infections, such as asthma [36], chronic obstructive pulmonary disease (COPD) [37] and cystic fibrosis [38,39].

Nevertheless, most of the currently available knowledge about the HRT microbiota is based on 16S rRNA gene sequencing analysis, thus restricting their taxonomic accuracy down to mostly genus level [40,41]. Moreover, currently published information pertaining to the HRT microbiota is generally aimed at identifying correlations between the HRT microbiota composition and HRT-associated diseases, thus targeting specific compartments of the respiratory tract, such as nasal cavity, larynx, or lung [21,42–46]. HRT surrogates, such as induced sputum and sputum, were also used to evaluate the upper and/or lower respiratory tract microbiota [29,47,48]. However, profiling of the lower respiratory tract microbiota through sputum and induced sputum samples may lead to taxonomical bias due to contamination of bacteria from the upper respiratory tract [49].

In order to provide a comprehensive view of the taxonomic composition of the HRT microbiota down to species level and to identify HRT-specific Community State Types (CSTs), we performed an in‐depth meta‐analysis of 16 publicly available shotgun metagenomic datasets corresponding to 849 samples obtained through different collection methods, i.e. lavage, swab, biopsy, and sputum, from healthy and diseased subjects [50–59]. Furthermore, the shotgun metagenomic data sets included in this study were further examined in order to dissect the genetic repertoire and microbial metabolic potential associated with such predicted HRT-CSTs.

## Materials and methods

### Database selection

In this meta-analysis-based study, we retrieved 16 publicly available data sets from studies involving the taxonomic determination of the human respiratory tract microbiome, performed in accordance with the relevant guidelines and regulations. In order to reduce the variability of the input data, we selected shotgun metagenomic datasets obtained by an Illumina sequencing platform. In detail, we selected shotgun metagenomic data sets from 849 samples from healthy or diseased subjects covering eight geographical regions (Table 1). The retrieved samples represented different collection methods, i.e. lavage, swabs, biopsies and sputum, from different HRT compartments (Supplementary Table S1).

**Table 1.** Metadata of the samples included in the meta-analysis

Bioproject	PMID	Nation	No. of samples	Nasal lavage	Nasopharynx swab	Sputum	Cough swabs	Oropharynx swab	Lung lavage	Biopsy	Undefined Swab
PRJEB28158	-	Germany	207	86	-	55	66	-	-	-	-
PRJEB9034	26,872,143	UK	18	-	-	18	-	-	-	-	-
PRJNA71831	28,158,639	USA	4	-	-	4	-	-	-	-	-
PRJNA644285	33,262,957	Brazil	3	-	-	3	-	-	-	-	-
PRJNA655567	-	China	61	-	-	-	-	-	53	-	8
PRJNA659860	-	Russia	14	-	-	-	-	-	14	-	-
PRJNA682527	-	Russia	25	-	-	-	-	-	-	25	-
PRJNA258008	-	USA	14	-	-	-	-	-	13	1	-
PRJNA316056	28,758,937	Italy	12	-	-	12	-	-	-	-	-
PRJNA316588	28,187,782	Switzerland	18	-	-	18	-	-	-	-	-
PRJNA494034	32,580,896	USA	12	-	-	-	-	-	12	-	-
PRJNA510441	-	USA	1	-	-	1	-	-	-	-	-
PRJNA413615	31,367,746	China	334	-	42	-	-	246	46	-	-
PRJNA516870	32,635,564	Italy	22	-	-	22	-	-	-	-	-
PRJNA516442	30,784,601	USA	11	-	-	11	-	-	-	-	-
PRJEB38221	33,319,812	Germany	93	-	-	-	93	-	-	-	-

### Taxonomic classification of sequence reads

Taxonomic profiling of sequenced reads was performed employing the METAnnotatorX2 bioinformatics platform [60,61]. In detail, the downloaded fastq files were filtered to remove reads with a quality of <25, and to retain reads with a length of  >100 bp. Subsequently, a human host DNA filtering was performed through bowtie2 software [62,63], following the METAnnotatorX2 manual [61]. Afterwards, taxonomic classification of 100,000 reads was achieved by means of MegaBLAST [64] employing a manually curated and pre-processed database of genomes retrieved from the National Center for Biotechnology Information (NCBI), following the METAnnotatorX2 manual [61].

### Functional prediction

Functional profiling of the sequenced reads was performed with the METAnnotatorX2 bioinformatics platform [60,61]. Functional classification of reads was performed to reveal metabolic pathways based on the MetaCyc database (release 24.1) [65] through RAPSearch2 software [66,67].

### Human respiratory tract Community State Type (HRT-CST) prediction

The HCL of samples was obtained using bacterial composition at species level and was calculated through ORIGIN 2021 (https://www.originlab.com/2021) software using Pearson correlation as a distance metric based on information at species level. The data obtained was represented by a cladogram.

### Statistical analysis

ORIGIN 2021 (https://www.originlab.com/2021) and SPSS software (www.ibm.com/software/it/analytics/spss/) were used to compute statistical analyses, including HCL and Silhouette analyses. EMPeror tool was used to visualize PCoA analyses [68] calculated through ORIGIN 2021. PERMANOVA analyses were performed using 1,000 permutations to estimate p-values for differences among populations in PCoA analyses. Furthermore, differential abundance of bacterial genera was tested by t-test or Mann–Whitney *U* test analysis. Multiple comparison analyses were performed through Tukey’s HSD (honestly significant difference) test. Moreover, multivariate analyses were performed through MaAsLin2 software [69].

## Results

### Dataset selection

In order to retrieve all publicly available metagenomic data sets concerning studies related to HRT microbiota, an extensive scientific literature search was performed (Figure S1). The scientific literature examination allowed us to collect HRT microbiota data from 16 publicly available data sets based on Illumina shotgun metagenomic methodologies, encompassing individuals from eight different countries (Figure S1, Table 1, and Supplementary Table S1). In detail, the multi-population cohort meta-analysis presented in this study includes datasets corresponding to a total of 849 samples from healthy or diseased subjects, obtained through different collection methods, i.e. lavage, swab, biopsy, and sputum (Table S1).

### Identification of human respiratory tract Community State Types (HRT-CSTs)

A total of 849 publicly available shotgun metagenomic datasets representing the HRT microbiota, including upper and lower respiratory tract, were collected. As reported in previous studies regarding human-associated microbiota, we employed a large number of HRT samples to aim for robust statistical accuracy [17,70–72]. In detail, we focused on samples obtained through Illumina shotgun sequencing to accurately profile bacteria at species level through re-analysis with the METAnnotatorX2 platform [60,61].

**Figure 1.**
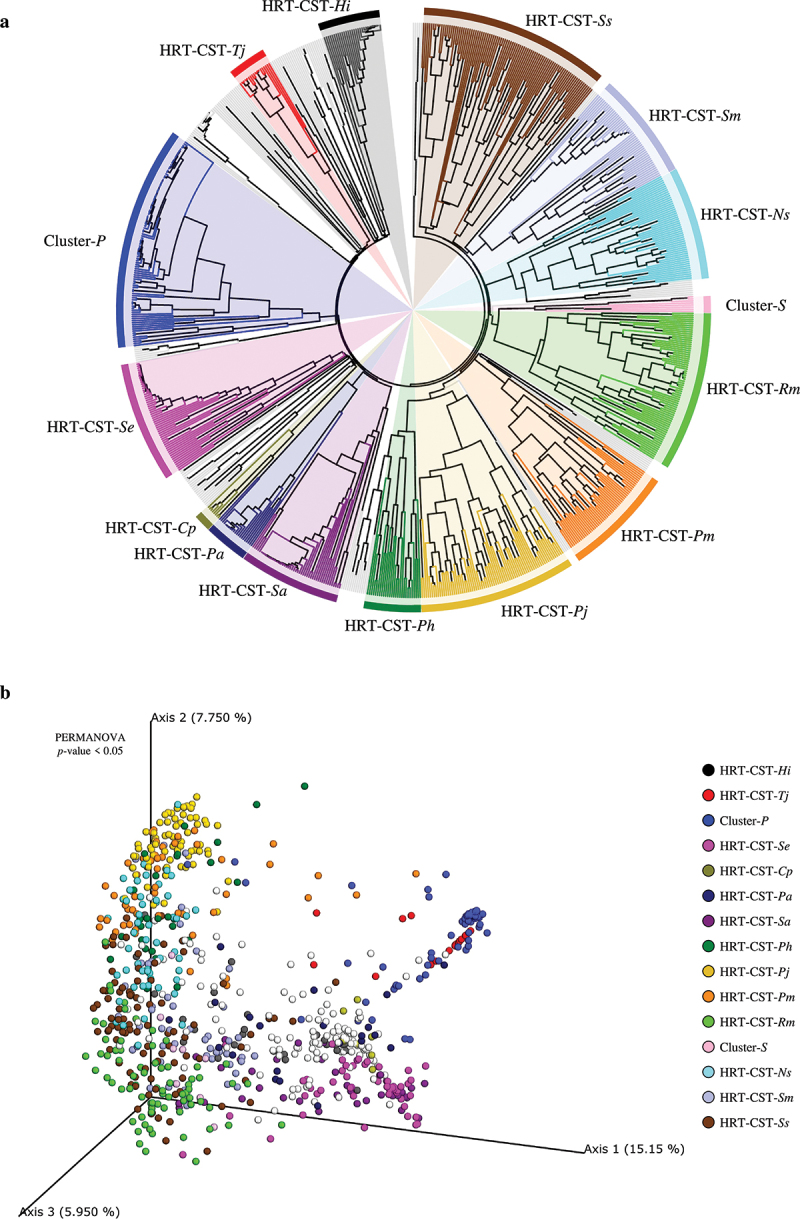
Identification of HRT-CSTs. Panel a shows a circular cladogram of the HRT samples obtained by means of hierarchical clustering (HCL) analysis. The cladogram highlights the different CSTs identified by HCL analysis. Panel b reports the principal coordinate analysis (PCoA) of the HRT samples, subdivided by identified CSTs.

Quality filtering and human DNA read-removal were performed starting from the collected fastq files and resulting in a total of 432,176 Mbp with an average of 1,002 ± 1,682 Mbp per sample (Table S1). The collected data sets were employed to predict the existence of common HRT-associated taxonomic profile patterns, leading to the identification of so-called HRT-CSTs. Identification of the minimal number of clusters necessary to define such HRT-CSTs was achieved by an unsupervised Silhouette method, revealing an optimal number of seven clusters (Figure S2a). Subsequently, a supervised cluster analysis through Hierarchical CLustering (HCL), involving the microbial taxonomic profiles at species level of HRT samples (Figure 1(a)), was performed to implement and biologically verify the results of the unsupervised approach, while also being supported by 3D Bray Curtis PCoA (Figure 1(b)). In detail, putative HRT-CSTs were defined by clusters represented by at least 1% of the total sample number, i.e. eight samples, and by samples from at least two different datasets to exclude possible biases related to a single study, as previously reported [73]. The validity of identified clusters was statistically confirmed by PERMANOVA (*p*-value < 0.05) based on PCoA analysis (Figure 1(b)). Furthermore, in order to exclude possible extrinsic bias, PCoA analyses were performed to identify possible associations between microbiota composition of each sample and the corresponding bioproject study or geographic origin (Figure S2a and b), revealing the absence of specific cluster and an heterogeneous distribution of samples.

The metagenomic meta-analysis of HRT samples allowed us to identify a total of 13 distinct CSTs (Figure 1(a) and Table 2), mainly characterized by species belonging to the Actinobacteria, Bacteroidetes, Firmicutes, and Proteobacteria phyla. Moreover, each identified CST was defined by the most abundant taxa with a prevalence > 85% (Table 2), while the remaining bacteria identified for each CST were considered accessory taxa. In detail, the meta-analysis revealed that the most prevalent CST of the HRT was HRT-CST-*Ss* (represented by 11% of the total number of samples) and HRT-CST-*Rm* (9% of the total sample number), which are dominated by *Streptococcus salivarius* and *Rothia mucilaginosa*, respectively (Table 2). Besides, five HRT-CSTs characterized by species belonging to the *Staphylococcus* and *Prevotella* genera were shown to be present at a prevalence ranging from 9% to 3%, and were defined as HRT-CST-*Se*, HRT-CST-*Sa*, HRT-CST-*Pj*, HRT-CST-*Pm* and HRT-CST-*Ph*, being characterized by a high relative abundance of *Staphylococcus epidermidis, Staphylococcus aureus, P. jejuni, P. melaninogenica* and *P. histicola*, respectively (Table 2). Furthermore, the meta-analysis allowed us to distinguish seven HRT-CSTs with a low prevalence (≤ 6% of the total sample) and typified by the presence of bacterial species considered opportunistic pathogens (Table 2). In detail, the latter were characterized by *Neisseria subflava* (HRT-CST-*Ns), Streptococcus mitis* (HRT-CST-*Sm), Haemophilus influenzae* (HRT-CST-*Hi), Tetrasphaera japonica* (HRT-CST-*Tj), Pseudomonas aeruginosa* (HRT-CST-*Pa*), and *Corynebacterium propinquum* (HRT-CST-*Cp*) (Table 2). Additionally, the HCL analysis revealed two clusters representing putative CSTs characterized by an unknown species belonging to the *Pasteurella* genus (Cluster-*P*) or *Schaalia* genus (Cluster-*S*) (Figure 1(a)). The predominance of sequences not associated with defined bacterial species indicates a non-specific classification with the consequent definition of putative HRT-CSTs, which are not associated with the presence of particular species. In order to define possible representative species of these two putative clusters, de novo assemblies through METAnnotatorX2 platform [60,61] were performed. Nevertheless, the high contamination of eukaryotic DNA and the low amount of bacterial reads did not allow to obtain reliable results. Moreover, Cluster-*S* appears to be characteristic of only one specific bioproject, i.e. PRJNA413615. For these reasons, Cluster-*P* and Cluster-*S* were excluded from HRT-CSTs classification and subsequent analyses. In this context, the biopsy samples displayed the lowest number of microbial profiles at a taxonomic resolution down to species level, being mainly represented by unknown species of *Pasteurella, Pseudomonas*, and *Tetrasphaera* genera. Consequently, due to the low number of samples and being derived mainly from a single bioproject (Table 1), biopsy samples were excluded from further analyses regarding the correlation between microbiota composition and anatomical body sites. Indeed, further studies involving a higher number of biopsy samples are needed to extend our comprehension of the microbial communities that adhere to pulmonary tissues.

**Table 2.** Average abundance and prevalence of bacteria that correspond to an identified HRT-CST

HRT-CSTs	Prevalence over the total		*Streptococcus salivarius*	*Prevotella jejuni*	*Rothiamucilaginosa*	*Staphylococcus epidermidis*	*Neisseria subflava*	*Prevotella melaninogenica*	*Streptococcus mitis*	*Staphylococcus aureus*	*Haemophilus influenzae*	*Prevotella histicola*	*Pseudomonas aeruginosa*	*Tetrasphaera japonica*	*Corynebacterium propinquum*
HRT-CST-*Ss*	11%	Average	10.43%	0.81%	5.59%	0.49%	0.54%	3.06%	3.51%	1.12%	0.25%	2.05%	0.75%	0.21%	0.08%
		Prevalence	92%	41%	92%	5%	19%	63%	79%	11%	9%	51%	10%	7%	2%
HRT-CST-*Pj*	9%	Average	2.68%	9.11%	1.10%	0.00%	2.29%	8.95%	2.52%	0.11%	0.03%	6.37%	0.00%	0.01%	0.00%
		Prevalence	81%	100%	64%	0%	73%	100%	84%	3%	1%	95%	0%	1%	0%
HRT-CST-*Rm*	9%	Average	2.61%	0.26%	28.08%	0.21%	3.02%	1.40%	3.17%	0.76%	0.18%	0.95%	0.67%	0.13%	0.03%
		Prevalence	69%	16%	100%	4%	50%	54%	66%	9%	5%	32%	7%	8%	1%
HRT-CST-*Se*	7%	Average	1.09%	0.03%	0.65%	50.12%	0.17%	0.09%	1.46%	2.70%	0.57%	0.03%	0.90%	1.92%	0.24%
		Prevalence	23%	4%	18%	100%	7%	7%	30%	29%	18%	2%	25%	41%	5%
HRT-CST-*Ns*	6%	Average	1.17%	1.78%	3.80%	0.00%	9.26%	6.53%	3.50%	0.07%	0.02%	1.17%	0.00%	0.13%	0.00%
		Prevalence	48%	65%	96%	0%	100%	100%	87%	4%	2%	52%	0%	8%	0%
HRT-CST-*Pm*	6%	Average	2.89%	2.09%	2.59%	0.03%	2.72%	18.45%	3.12%	0.75%	0.07%	4.20%	0.03%	0.42%	0.00%
		Prevalence	63%	88%	79%	2%	73%	100%	79%	4%	6%	71%	2%	12%	0%
HRT-CST-*Sm*	6%	Average	1.56%	1.02%	2.22%	0.31%	1.54%	2.38%	22.59%	0.03%	1.13%	0.86%	0.00%	0.11%	0.00%
		Prevalence	60%	28%	74%	4%	40%	70%	100%	4%	30%	28%	0%	10%	0%
HRT-CST-*Sa*	6%	Average	1.14%	0.10%	1.44%	1.33%	0.07%	0.40%	1.49%	59.58%	0.29%	0.42%	2.27%	0.96%	0.43%
		Prevalence	33%	6%	38%	25%	4%	19%	33%	100%	8%	8%	27%	17%	4%
HRT-CST-*Hi*	3%	Average	2.56%	0.59%	1.20%	1.64%	0.53%	0.91%	2.93%	0.85%	51.04%	1.85%	0.00%	0.74%	0.35%
		Prevalence	59%	31%	45%	17%	24%	48%	59%	7%	100%	38%	0%	31%	3%
HRT-CST-*Ph*	3%	Average	2.93%	2.79%	1.76%	0.05%	1.15%	5.99%	3.10%	0.07%	0.15%	16.42%	0.06%	0.18%	0.00%
		Prevalence	70%	74%	74%	4%	44%	93%	74%	4%	4%	100%	4%	15%	0%
HRT-CST-*Pa*	2%	Average	1.28%	0.41%	1.97%	0.00%	0.00%	0.56%	0.80%	2.14%	0.00%	0.63%	55.84%	2.37%	0.00%
		Prevalence	24%	19%	38%	0%	0%	29%	29%	24%	0%	24%	100%	38%	0%
HRT-CST-*Tj*	2%	Average	0.00%	0.84%	0.00%	0.00%	0.25%	1.94%	1.59%	0.00%	0.00%	0.00%	0.15%	41.09%	0.00%
		Prevalence	0%	11%	0%	0%	6%	22%	11%	0%	0%	0%	6%	100%	0%
HRT-CST-*Cp*	1%	Average	0.42%	0.00%	0.08%	2.04%	1.69%	0.00%	0.00%	2.63%	1.42%	0.00%	0.00%	4.24%	50.97%
		Prevalence	13%	0%	13%	38%	25%	0%	0%	25%	13%	0%	0%	63%	100%

In red are highlighted the relative abundances > 9%, while in green are selected prevalence > 85%

### Correlation between HRT microbiota and human body-anatomical regions

The identification of specific HRT-CSTs representative of the respiratory tract as a single compartment suggested to investigate possible correlations between the specific respiratory compartments, such as lung, throat and nasal cavity, and the HRT-CSTs. In detail, the high number of samples included in the meta-analysis allowed us to explore possible correlations between the HRT microbiota composition and different sampling methods, i.e. lavage, swab, and sputum.

**Figure 2.**
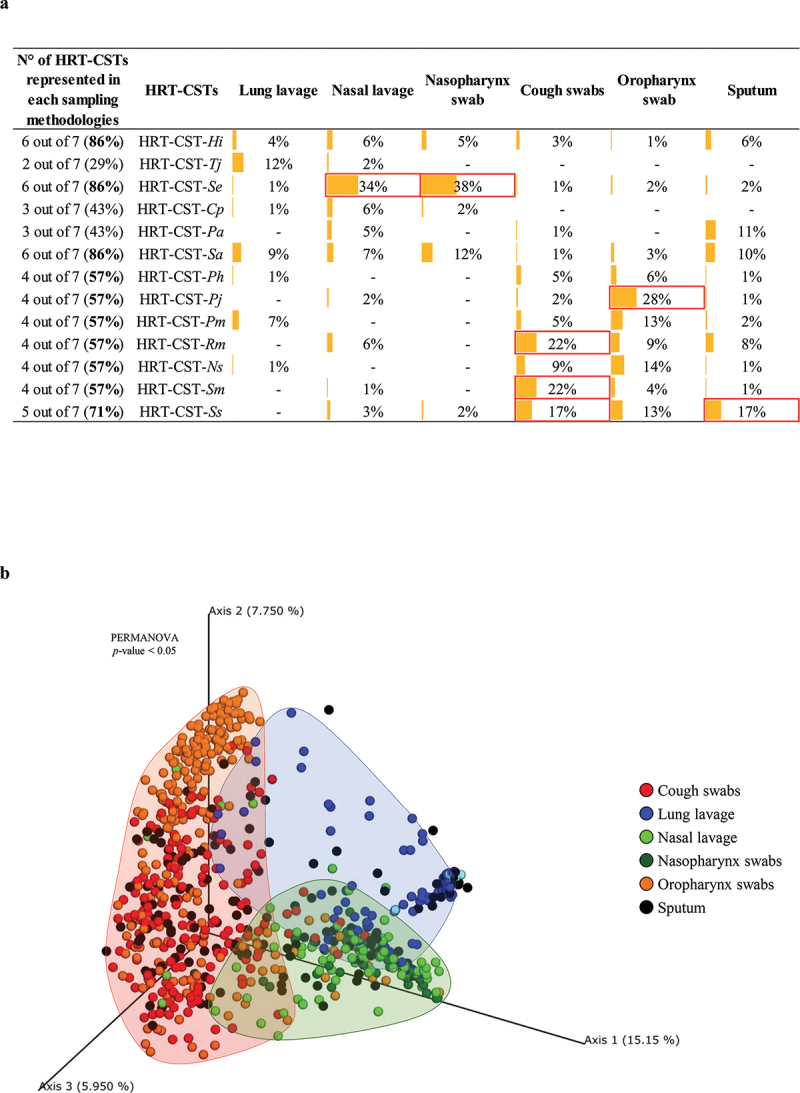
Evaluation of HRT-CST distribution based on sampling methods. Panel a shows the correlations between HRT-CST and the different sampling methods. Prevalences > 15% were highlighted in red. Panel b displays the principal coordinate analysis (PCoA) of the HRT samples, subdivided by sampling methods.

Analysis of HRT-CSTs’ prevalence showed that all HRT-CSTs encompass samples of at least two different sampling methodologies and represent a common microbial community profile of distinct compartments of the respiratory tract (Figure 2(a)) [32,33]. Moreover, samples from the nasal cavity, i.e. nasal lavage and nasopharynx swab, were shown to exhibit a high prevalence of HRT-CST-*Se* (prevalence of 34% and 38%, respectively). Furthermore, the analyzed oropharynx samples showed a high prevalence of HRT-CST-*Pj* (28%), while cough swabs were demonstrated to exhibit a high prevalence of HRT-CST-*Rm* (22%) and HRT-CST-*Sm* (22%) (Figure 2(a)). In contrast, sputum and lung lavage samples revealed a variable microbiota composition, showing the absence of any specific HRT-CST related to any of these two compartments (Figure 2(a)). Analysis of the beta-diversity represented through Bray Curtis 3D-PCoA (Figure 2(b)) revealed three specific clusters independent of the sampling methods but related to particular respiratory tract compartments, i.e. nasal cavity, throat, and lung, thus indicating taxonomic features specific to each of these anatomic regions PERMANOVA (*p*-value < 0.05). Conversely, sputum samples elicited a heterogeneous microbial distribution and therefore a more variable sputum microbiota composition (Figure 2(b)).

**Figure 3.**
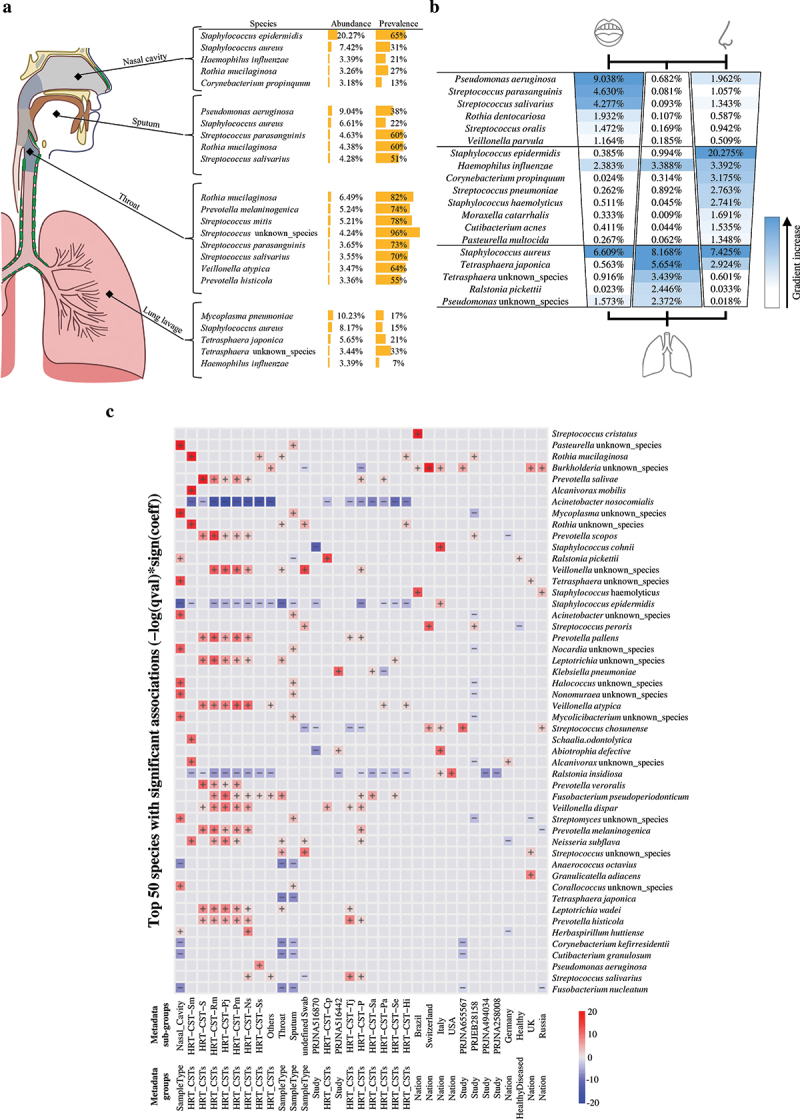
Representation of the main microbial taxa characterizing the different anatomical regions of HRT. Panel a indicates the most abundant bacterial species for each compartment of the human respiratory tract. Panel b reports the taxa showing a possible abundance gradient in relation to the distance from oral, lung and nasal compartments, respectively. Panel c shows an heatmap of the specific multivariate analysis through MaAslin2 software based on sampling methods as well as HRT-CSTs as well as bioproject, and geographical origin. Only the top 50 species with significant associations are reported. Significant positive correlations are reported in red, while Significant negative correlations are reported in blu.

Consequently, we decided to investigate the presence of specific bacterial communities associated with specific HRT compartments. In detail, we focused on 53 bacterial species with a relative abundance > 1% in at least one compartment group (Table S2). In detail, nasal cavity, throat, and lung compartments shared a total of 40 microbial species (Table S2), highlighting common microbial community inhabitants of different compartments of the respiratory tract. Moreover, samples from the nasal cavity presented a high prevalence and relative abundance of *Staphylococcus epidermidis* (Table S2 and Figure 3(a)). It is possible that *Staphylococcus epidermidis* as a common component of the nasal microbiome plays a major role in promoting ecological competition between commensal bacteria and opportunistic pathogenic species, as reported previously [74,75]. Interestingly, the presence of skin-colonizing bacteria such as *Staphylococcus epidermidis* indicates a relationship between the microbiota of the nasal cavity and that of the skin, suggesting a bacterial transition between these ecological niches. Additionally, in the other assessed HRT compartments, i.e. throat and lung, *Staphylococcus epidermidis* is present at a lower relative abundance than the nasal cavity, probably reflecting a decreasing abundance gradient based on distance from the nasal cavity (Figure 3(b)). Besides, seven other species, including *Haemophilus influenzae* and *Streptococcus pneumoniae*, are shared among the different body-compartments and were shown to be present at a decreasing abundance gradient depending on the distance from the nasal cavity (Figure 3(b)). Intriguingly, a specific multivariate analysis through MaAslin2 software [69] and based on sampling methods, HRT-CSTs, bioproject, and geographical origin, revealed that among the taxa characterizing each CST, *S. epidermidis, H. influenzae* and *Str. pneumoniae* possessed the largest number of significant negative associations (all false discovery rate (FDR) < 0.05) with the HRT-CSTs (Figure 3(c) and Table S3). These results could suggest an high ability of these bacterial species to compete for their specific ecological niche. Furthermore, throat, i.e. cough and oropharynx swabs, sputum and lung lavage samples were shown to exhibit heterogeneous microbial compositions, without a predominant taxon, perhaps representing a transitory microbiota. Intriguingly, the throat microbiota appears to be characterized by species belonging to the genera *Streptococcus* and *Rothia* [76], such as *Streptococcus parasanguinis, Streptococcus salivarius* and *R. mucilaginosa*, which are also representatives of the sputum microbiota (Table S3), indicate a tight transition of bacteria between these two body-compartments. In accordance with these results, the multivariate analysis of these taxa revealed a limited number of significant associations, probably indicating these species as components of a transient microbiota (Table S3). In addition, sputum and lung lavage samples revealed a specific taxonomical gradient based on distance from each compartment (Figure 3b). In detail, lung lavage samples showed five bacterial species, such as *Tetrasphaera japonica*, with a higher relative abundance compared to other compartments, indicating a possible increasing gradient (Figure 3(b)). In contrast, sputum samples revealed six species, represented by *Pseudomonas aeruginosa, Streptococcus parasanguinis, Streptococcus salivarius, Streptococcus oralis, Rothia dentocariosa*, and *Veillonella parvula*, with a distance-dependent gradient from the oral cavity, possibly reflecting bacterial adaptation to a specific ecological niche.

### Association between HRT-CSTs and respiratory tract disease

**Figure 4.**
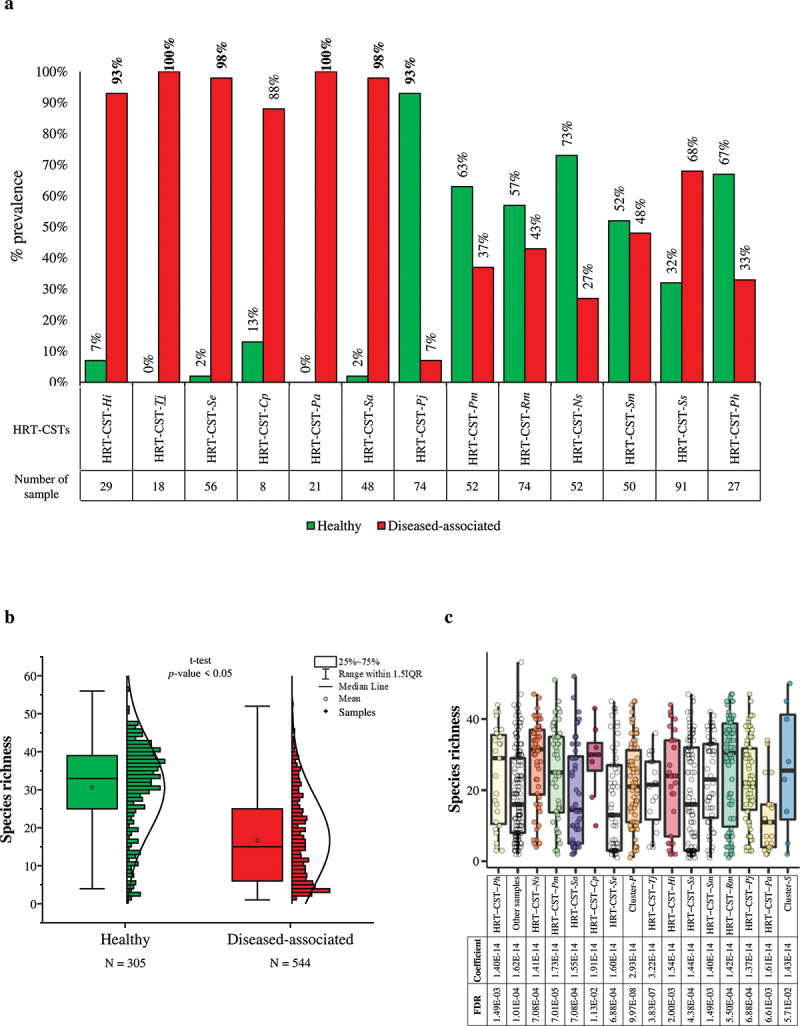
Evaluation of the correlations between HRT-CSTs and respiratory tract diseases. Panel a indicates possible correlations between HRT-CST and the disease, reporting the prevalence values of each HRT-CSTs. Panel b displays the Whiskers plot representing the species richness identified from healthy and disease-associated samples. The x‐axis represents the different groups, while the y‐axis indicates the number of species. The boxes are determined by the 25^th^ and 75^th^ percentiles. The whiskers are determined by 1.5 IQR (Interquartile range). The line in the boxes represented the median, while the square represents the average. Panel c reports the multivariate analysis calculated through MaAslin2 software and based on species richness, as well as HRT-CSTs, sampling methods, bioproject, and geographical origin.

The samples involved in this meta-analysis include both healthy and diseased samples (Table S1). In fact, the main purpose of this study was to assess the HRT microbiota regardless of host health status. Nevertheless, we explored the statistical power of this meta-analysis to identify correlations between HRT-CSTs and respiratory tract disease. Unfortunately, the absence of comprehensive metadata regarding the classification of the pulmonary pathology prevented detailed comparisons between healthy and diseased samples and identifying the possible correlations between HRT microbiota composition and specific pulmonary diseases. In detail, this analysis highlighted that diseased samples appeared to be associated with five HRT-CSTs, i.e. HRT-CST-*Hi*, HRT-CST-*Tj*, HRT-CST-*Se*, HRT-CST-*Pa*, and HRT-CST-*Sa*, with a prevalence > 90% (Figure 4(a)). In contrast, only HRT-CST-*Pj* is associated with healthy samples (prevalence = 93%) (Figure 4(a)). Furthermore, analysis of species richness revealed a lower complexity of the diseased samples (species richness of 31 ± 12) compared to samples obtained from healthy subjects (species richness of 17 ± 12) (*p*-value < 0.05) (Figure 4(b)). These data indicate a correlation between simplification of the HRT microbiota and (the onset of) respiratory disorders, possibly due to the predominance of opportunistic bacteria, such as *Haemophilus influenzae, Pseudomonas aeruginosa*, and *Staphylococcus aureus* [77–79]. In contrast, healthy samples possess a more heterogeneous microbiota with higher microbial biodiversity, confirming the concept that healthy subjects possess a more diverse bacterial HRT community that is considered to enjoy homeostasis [38]. Moreover, a specific multivariate analysis through MaAslin2 software [69] based on sampling methods as well as HRT-CSTs as well as bioproject, and geographical origin, revealed that the main significant correlations are positive and related to HRT-CSTs but with low model coefficient values (Figure 4(c) and Table S4), suggesting an absence of correlation with the HRT community state types. Notably, the multivariate analysis highlighted the absence of correlations with the health status of the respiratory tract, but this result could be influenced by the type of distribution of the diseased samples (Figure 4(b)). Certainly, further analyses focused on the investigation of bacterial differences between different respiratory tract diseases and healthy individuals are necessary to elucidate the role and/or the association of the HRT with the onset of respiratory pathologies.

### Functional capabilities based on anatomical human body regions

**Figure 5.**
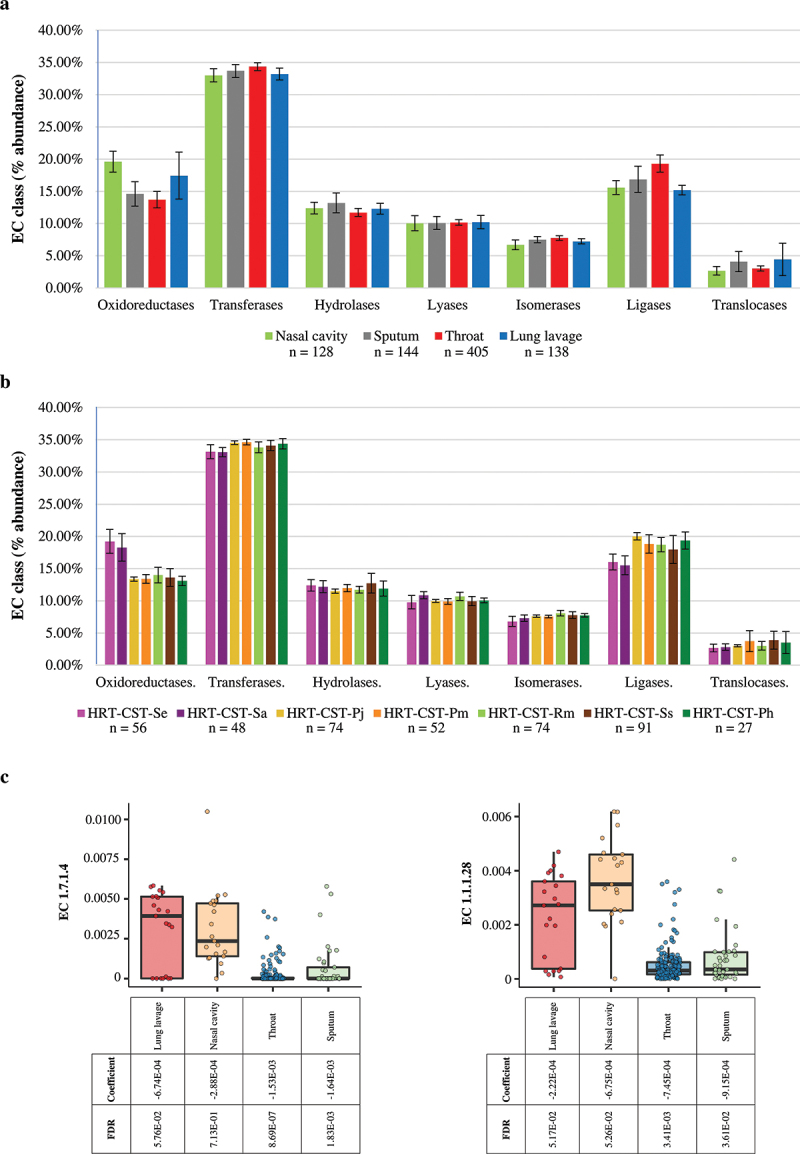
Evaluation of metabolic pathways of HRT samples. Panel a reports on the bar plot representing the EC class abundance based on HRT compartments. The x‐axis represents the different HRT compartments, while the y‐axis indicates the relative abundance of each EC class. The whiskers reported the standard deviation. Panel b shows the bar plot indicating the EC class abundance based on HRT-CSTs. The x‐axis represents the different HRT compartments, while the y‐axis indicates the relative abundance of each EC class. The whiskers report the standard deviation. Panel c reports the multivariate analysis calculated on the two enzyme classes EC 1.7.1.4 and EC 1.1.1.28 through MaAslin2 software and represented with bar plot. The MaAsLin2 model are fitted with the enzyme classes, as well as HRT-CSTs, sampling methods as well as bioproject, and geographical origin.

The specific microbial community profiles of different body compartments are assumed to correspond to specific microbiomes and genetic repertoires. In order to explore the genetic features characterizing each HRT compartment, we performed a screening of metabolic pathways based on the MetaCyc database [65]. This metabolic analysis included all HRT samples except biopsy samples. In fact, biopsy samples after human-DNA filtering did produce enough microbial DNA data to perform the metabolic analysis. Therefore, evaluation of enzyme classes based on Enzyme Commission (EC) number revealed differences in the relative abundance of predicted enzyme functions between different respiratory compartments. In detail, samples from the nasal cavity and lung lavage showed the highest abundance of oxidoreductases (average abundance 19.60% ± 1.62% and 17.43% ± 3.65%, respectively) when compared to other HRT compartments (ANOVA *p*-value < 0.01, Tukey’s HSD post-hoc test *p*-value < 0.01) (Figure 5(a)). Similarly, analysis based on HRT-CSTs with a prevalence of > 3% revealed that HRT-CST-*Se* and HRT-CST-*Sa*, being the main representatives of the nasal tract and lung lavage, respectively, reflected the enzyme class abundance-trend of the respiratory tract compartments (Figure 5(b)). These results highlight a metabolic adaptation by bacterial communities based on their ecological niche and/or type of sampling. In detail, nasal cavity and lung lavage samples revealed a total of 21 EC oxidoreductase sub-classes with the highest significant relative abundance compared to other HRT compartments (Tukey’s HSD post-hoc test p-value < 0.01), including enzymes predicted to be responsible for formation of nitric oxide, e.g. nitrite reductase (EC 1.7.1.4) and nitric-oxide synthase (EC 1.14.13.39), and in the production of lactate, e.g. D- and L-lactate dehydrogenase (EC 1.1.1.28 and 1.1.1.27, respectively) (Table S5). These results are confirmed by the multivariate analysis calculated through MaAslin2 software [69], which revealed a significant negative correlation between EC 1.7.1.4 and 1.1.1.28 with sputum and throat samples (Figure 5(c) and Table S6). In detail, nitric oxide is reported to exert an important physiological role in the regulation of pulmonary vasomotor tone [80,81] and an increase of enzymes involved in this metabolic pathway validates the mutualistic interaction between HRT microorganisms and their host. Moreover, the predicted higher abundance of enzymes involved in lactate production indicates the importance of certain bacteria in keeping the nasal environment at a relatively low pH, thereby supporting antimicrobial effects [82]. Furthermore, focusing on EC sub-classes specific for each HRT compartment, lung lavage samples showed an increase of 155% of Cd(2+)-exporting ATPase (EC 7.2.2.21) when compared to other HRT compartments (Tukey’s HSD post-hoc test p-value < 0.01) (Table S5). This enzyme is involved in heavy metal detoxification [83], and its relatively high abundance in the lung suggests an ecological adaptation of bacteria to the pulmonary environment, perhaps due to the continuous intake of air pollutants [84,85]. In addition, sputum and throat samples revealed a higher abundance of enzymes involved in carbohydrate metabolism, e.g. glycogen phosphorylase (EC 2.4.1.1), alpha-amylase (EC 3.2.1.1), pullulanase (EC 3.2.1.41), and dextranase (EC 3.2.1.11), compared to other compartments, suggestive of an adaptation of bacterial communities to the oral environment that is involved in preliminary digestive processes [86,87]. The small presence of these enzymes in the others anatomic sites of lower respiratory tract could be explained by a partial contamination/transition of bacteria characterizing the oral microbiota to the sputum and throat compartments [43], but also could indicate a possible specific selective pressure from the specific respiratory anatomic sites [88].

An extensive analysis based on metabolic pathways prediction confirmed possible differences in relation to the different HRT compartments. In detail, the sputum samples presented a higher number of unique pathways, i.e. 237 pathways, respect to the throat samples, nasal cavity, and lung lavage that displayed 101, 86, and 95 unique pathways, respectively. Similarly, focusing on the degradation and biosynthesis pathways, the throat, nasal cavity, and lung lavage samples highlighted a similar number of unique pathways respect to sputum samples showed the highest number of unique pathways (Table S7). These results could confirm the adaptation of microbial communities to different ecological niches, highlighting the high metabolic commitment of the oral bacteria involved in several specific physiological activities, such as preliminary digestive processes, specific to HRT compartments.

## Discussion

The HRT harbors a complex community of microorganisms, that are believed to play a major role in preserving physiological homeostasis of the host [18,89]. However, in contrast to the gastrointestinal tract, which represents the most thoroughly investigated organ-microbiota niche, the human respiratory tract remains relatively poorly investigated [90,91]. Despite several studies based on culture-independent metagenomic analyses aimed at evaluating the microbiota composition of different HRT sites, a comprehensive meta-analysis is still missing. Here, we collected a total of 849 HRT samples from publicly available shotgun metagenomic datasets, representing the respiratory tract as a single compartment and facilitating an in‐depth meta‐analysis. This statistically robust meta-analysis allowed us to identify 13 possible HRT-specific Community State Types (CSTs), mainly characterized by species belonging to *Streptococcus, Staphylococcus, Prevotella, Neisseria*, and *Rothia* genera. Furthermore, analysis of the distribution of each HRT-CSTs along the respiratory tract highlighted a possible specific microbial correlation with the different HRT compartments. In this context, our meta-analysis highlighted a location-specific abundance gradient in HRT of certain bacterial species, such as *Staphylococcus epidermidis, Streptococcus salivarius*, and *Pseudomonas aeruginosa*, reinforcing the notion of a possible bacterial adaptation to a specific ecological niche within the HRT. Such findings were further corroborated by metabolic reconstruction of metagenomic datasets from different HRT regions. Intriguingly, samples from the nasal cavity and lung lavage compared to those obtained from other HRT compartments showed statistically significant differences in the predicted microbial enzyme profiles, including metabolic pathways involved in nitric oxide and lactate production. These results suggest the existence of a correlation between the different compartments of the respiratory tract and highlight the important role that the HRT microbiota plays in maintaining host homeostasis. Conversely, sputum samples revealed a higher abundance of enzymes implicated in carbohydrate metabolism, revealing a possible metabolic adaptation of resident bacteria to specific ecological niches. Certainly, specific *in vitro* metabolism studies will be useful to provide further data on the effective metabolic capabilities of each HRT-CST.

Moreover, the lack of complete public metadata, such as gender, age, and diet, did not allow us to perform in depth multiple correlations with the composition of the HRT-CSTs microbiome. Remarkably, the predominance of data derived from samples associated with lung diseases when compared to healthy samples of certain HRT compartments, i.e. lavage samples, and the lack of in depth details on the type of pulmonary pathology, e.g. disease severity, very much limited our ability to perform meaningful comparisons between healthy and diseased samples. In this context, specific studies assessing bacterial differences between different lung diseases and healthy individuals using shotgun metagenomics approaches are expected to contribute to the identification of biomarkers that are involved in or associated with the onset of HRT pathologies.

## Conclusion

In conclusion, our meta-analysis allowed the identification of 13 putative Community State Types (CSTs), which appear to correlate with the different HRT compartments. Moreover, several bacterial species display a location-specific abundance gradient in HRT, suggesting a possible bacterial adaptation to a specific HRT compartment. Furthermore, the metabolic reconstruction of HRT metagenomic datasets revealed significant differences in the predicted enzyme profiles, suggesting a potential role of the HRT microbiota in maintaining host homeostasis and confirming a possible metabolic adaptation of resident bacteria to specific ecological niches.

## Supplementary Material

Supplemental Material
