# Supplementary material for: Mapping bacterial diversity and metabolic functionality of the human respiratory tract microbiome
Source: J Oral Microbiol. 2022 Mar 16;14(1):2051336. doi: 10.1080/20002297.2022.2051336 (PMC8933033; doi:10.1080/20002297.2022.2051336)
Supplement: Supplemental Material [file ZJOM_A_2051336_SM1256.zip › Supplementary files/supplementary_information_2.docx]

**Mapping bacterial diversity and metabolic functionality of** **the human respiratory tract microbiome.**

Leonardo Mancabelli^1^, Christian Milani^1,2^, Federico Fontana^1^, Gabriele Andrea Lugli^1^, Chiara Tarracchini^1^, Francesca Turroni^1,2^, Douwe van Sinderen^3^ and Marco Ventura^1,2^

**Supplementary information**

**Additional files**

**Figure S1.** Flow diagram showing the primary details regarding sample selection and analysis.

**Figure S2.** Silhouette analysis and PCoA analyses based on bioproject study and geographic origin.

**Supplementary tables S1.** Studies included in this meta-analysis.

**Supplementary tables S2.** Evaluation of the bacteria shared between different HRT compartments.

**Supplementary tables S3.** Multivariate analysis based on taxonomy composition calculated through MaAslin2 software.

**Supplementary tables S4.** Multivariate analysis based on species richness calculated through MaAslin2 software.

**Supplementary tables S5.** Prediction of the metabolic pathways.

**Supplementary tables S6.** Multivariate analysis based on metabolic pathways calculated through MaAslin2 software.

**Supplementary tables S7.** Prediction of unique metabolic pathways.
