# Supplementary figures and images for: Mapping bacterial diversity and metabolic functionality of the human respiratory tract microbiome
Source: J Oral Microbiol. 2022 Mar 16;14(1):2051336. doi: 10.1080/20002297.2022.2051336 (PMC8933033; doi:10.1080/20002297.2022.2051336)

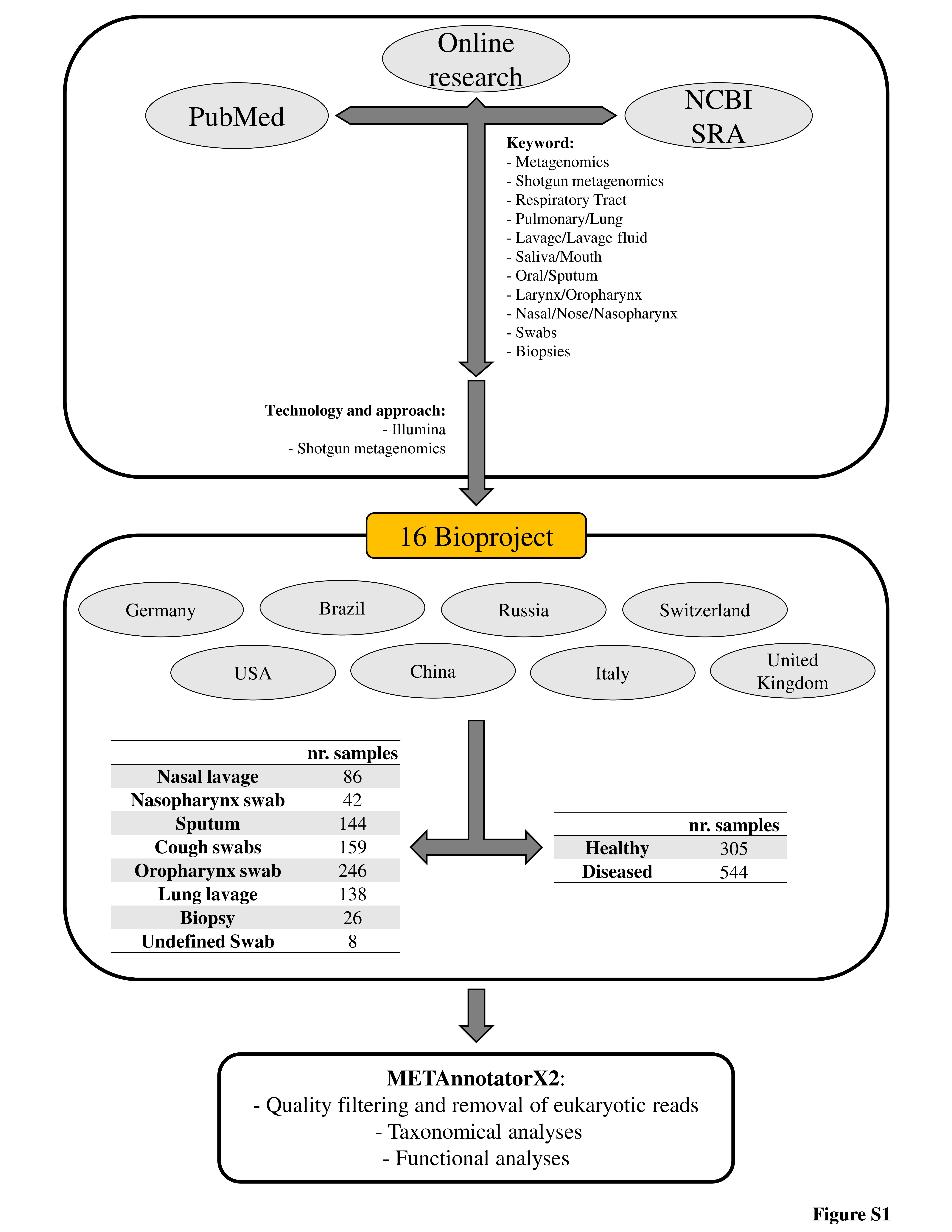

Supplement: Supplemental Material [file ZJOM_A_2051336_SM1256.zip › Supplementary files/Figure_S1_new.jpg]

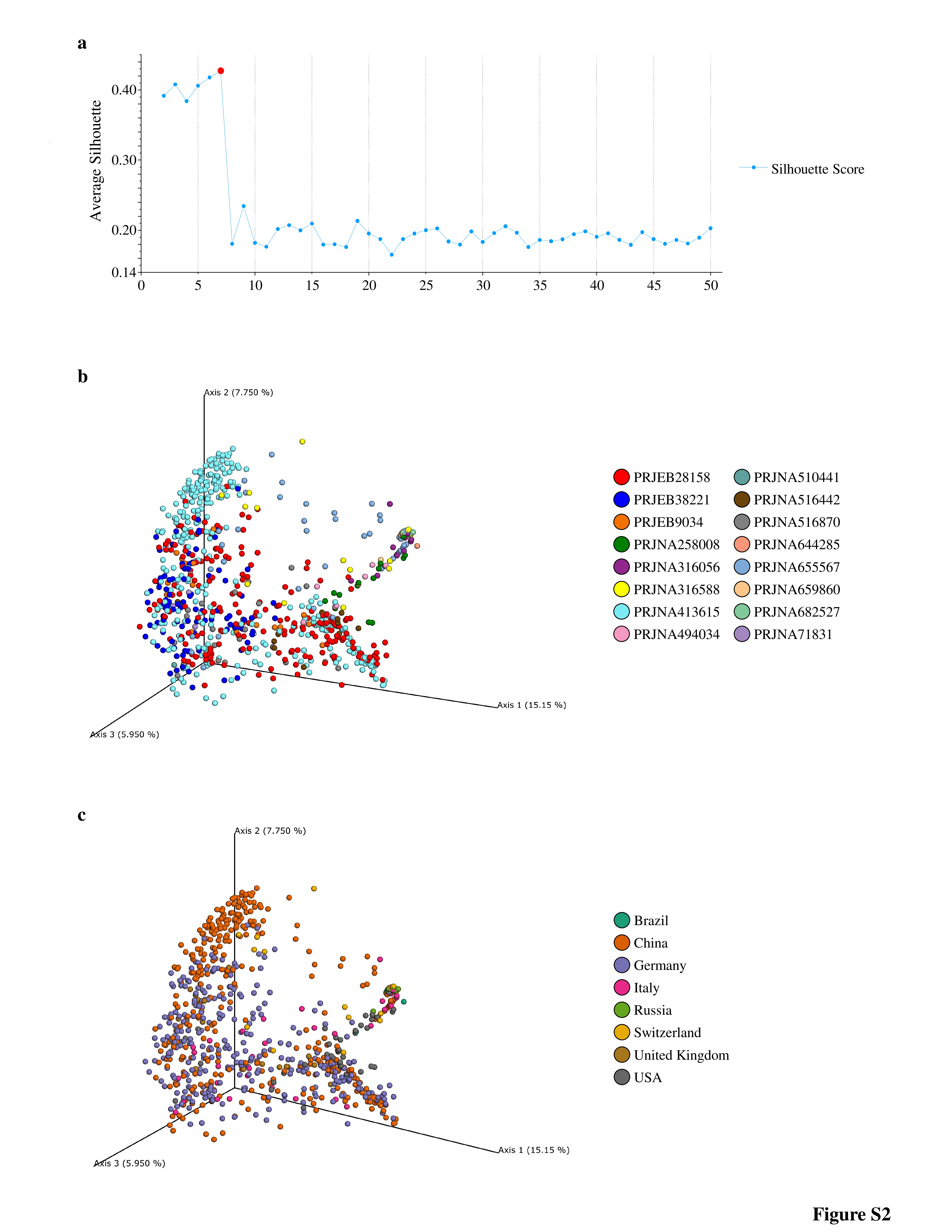

Supplement: Supplemental Material [file ZJOM_A_2051336_SM1256.zip › Supplementary files/Figure_S2_new_3.jpg]
